# Beta Blocker Intoxications in Belgium: A Data Analysis with Focus on Propranolol

**DOI:** 10.3390/pharmacy14020043

**Published:** 2026-03-04

**Authors:** Brechje van den Boogaard, Maria van de Lavoir, Rani Robeyns, Celine Gys, Adrian Covaci, Hans De Loof

**Affiliations:** 1 Toxicological Centre, University of Antwerp, Universiteitsplein 1, 2610 Antwerp, Belgium; brechjevandenboogaard@outlook.com (B.v.d.B.); annemiekevandelavoir@gmail.com (M.v.d.L.); rani.robeyns@uantwerpen.be (R.R.); celine.gys@uantwerpen.be (C.G.); adrian.covaci@uantwerpen.be (A.C.); 2 Laboratory of Physiopharmacology, University of Antwerp, Universiteitsplein 1, 2610 Antwerp, Belgium

**Keywords:** beta blockers, propranolol, intoxication, overdose, poisoning, pharmacovigilance

## Abstract

**Background:** The issue of beta blocker poisoning has received little attention, despite the widespread use of these compounds in cardiac and neuropsychiatric care. Safety profiles differ, and some beta blockers appear in poisonings far beyond what their usage rates imply. This study characterizes beta blocker intoxication patterns in Belgium, focusing on propranolol, by integrating national prescription data, poisoning reports, and adverse drug reaction records. **Methods:** Belgian prescription data, poison centre reports, and European ADR databases were analysed to identify intoxication patterns and demographic or clinical characteristics associated with these events. **Results:** Poisoning data revealed propranolol as markedly overrepresented compared to prescription rates and was the primary beta blocker implicated in self-harm-related overdoses. These cases occurred mainly in women, younger individuals, and patients with psychiatric or cardiovascular comorbidities. Co-exposures with benzodiazepines, antidepressants, and other psychoactive agents were frequent, and propranolol was linked to more complex intoxication patterns than other beta blockers. **Conclusions:** Propranolol shows a distinct toxicological profile and is disproportionately involved in intoxications, especially in vulnerable groups and in combination with psychoactive drugs. These findings highlight the need for greater awareness, targeted prevention, and careful monitoring.

## 1. Introduction

Beta blockers are a cornerstone in the treatment of cardiovascular diseases, with their development dating back to 1958 when propranolol was introduced as the first non-selective agent by Sir James Black [1,2]. This discovery was of such significance that Black was awarded the Nobel Prize in Medicine in 1988. Therapeutic options for angina were restricted to nitrates prior to propranolol, and these provided merely partial symptom control. Further progress led to cardioselective and vasodilating agents, improving safety and heart failure outcomes. Beta blockers remain a widely utilized class of synthetic agents in contemporary medicine, with several, including propranolol, designated as essential medicines by the WHO [1,3].

Despite their clinical benefits, beta blockers can cause serious toxic effects when overdosed and were identified as the 7th leading overall cause of poisoning deaths in the United States according to the 2020 National Poison Center Data System [4]. Data from the California Poison Control System indicate that antihypertensive overdoses, including beta blockers, often result in bradycardia, hypotension, respiratory distress, and cardiac arrest [5].

After oral ingestion, the first symptoms of poisoning typically appear within 1 to 2 h, with peak effects occurring up to 12 h later in cases involving extended-release formulations [6]. No specific lethal dose has been established, as the toxic threshold varies between individuals. Blood concentrations are of limited value in assessing the clinical presentation of beta blocker intoxication, since there is no clear correlation between plasma levels and clinical outcomes [7]. Therefore, clinical symptoms are the primary determinant. Patients taking additional cardiovascular medications, those with a history of heart disease, or those with impaired kidney function are at increased risk of more severe effects—even at lower doses [6]. Propranolol, compared to other beta blockers, is disproportionately associated with self-poisoning and fatalities, likely due to its frequent use in patients at higher risk for suicidal behaviour [8,9,10].

Propranolol is also a highly lipophilic beta blocker, with high membrane stabilizing activity (MSA), enabling it to easily cross cell membranes and the blood–brain barrier. In overdose, this can lead to neurological manifestations, such as seizures and delirium [4,11,12]. Its MSA allows inhibition of fast sodium channels, which may result in a prolonged QRS complex and further exacerbation [11,13,14].

Recent studies from the UK show a rising number of propranolol-related fatalities, especially among women and younger individuals [15,16,17,18,19]. Although propranolol has occasionally been linked to depression, the evidence is not definitive. Current findings point toward pre-existing mental health conditions rather than a direct pharmacological effect [20,21]. Given its frequent co-prescription with antidepressants, there is a need to better understand its combined toxicological profiles [15,16,17,18]. We performed a retrospective analysis comparing propranolol prescription data and that of other commonly used beta blockers with multiple sources of adverse effect reports and local toxicology records. Based on international evidence, we expect to observe a similar pattern in Belgium, with propranolol appearing disproportionately often in poisoning reports despite its relatively low prescription volume.

## 2. Materials and Methods

This study integrates national usage data from the Belgian Poison Centre and Farmanet with toxicology databases, supplemented by European adverse drug reaction (ADR) data from EudraVigilance, to analyse beta blocker intoxication patterns, subgroup distributions, and recent trends in co-medication. Because the datasets differed in structure and completeness, we first converted all variables to a shared yearly format. Cases with missing essential demographic or exposure information were excluded. For comparisons across datasets, we used only variables that were available in all sources, such as year, substance, age group, and sex to ensure compatibility.

The various data and/or statistical analyses and the production of graphical illustrations were carried out with Python (Version 3.8.20) using the Scientific Python Development Environment (Spyder, Version 5.4.3) under Anaconda3 (Distribution 2023.09-0). This study used fully anonymized and publicly available data. According to applicable institutional and national regulations, research using such data does not require ethics committee review. Therefore, no ethics approval was required. Additional methodological details and extended visualizations are provided in the Supplementary Materials.

### 2.1. Usage of Beta Blockers

The usage of beta blockers in Belgium was analysed for the period 2013–2023, based on data provided by Farmanet [22]. This database contains information on medication dispensed by public pharmacies and reimbursed through the mandatory health insurance system. It provides reliable insights into pharmaceutical consumption in Belgium, enabling the identification of trends and patterns in beta blocker use. The analysis focused on two key yearly parameters: the number of unique patients (NUP), representing the count of individual patients who received a beta blocker, and the number of Defined Daily Doses, reflecting the total volume of consumption expressed in DDDs, based on the average adult maintenance dose for the drug’s main indication. The DDD is maintained by the WHO Collaborating Centre and offers a standardized measure that is independent of price, currency, and drug strength. It is used to harmonize variations in drug consumption, allowing for meaningful comparisons across regions and time periods [23].

Average medication use of all beta blocker patients was estimated using the DDDs/NUP ratio. Trends over time in DDDs, NUP, and DDDs/NUP for each beta blocker were assessed using Kendall’s tau, a non-parametric test for rank correlation, implemented through the Kendall Tau function in Python’s SciPy library. Tau values range from −1 (perfect decreasing trend) to 1 (perfect increasing trend), with 0 indicating no monotonic trend. The accompanying *p*-value tests whether the observed trend is statistically significant (α = 0.05). With this *p*-value a null hypothesis that the variables are independent can be tested. A small *p*-value (e.g., smaller than a prior chosen significance level α = 0.05) rejects the null hypothesis (no correlation) and indicates a significant correlation. This test allowed us to determine whether trends were increasing or decreasing and whether they were statistically significant. Kendall’s tau was chosen because it is non-parametric, robust to outliers, suitable for small samples, and does not assume linearity or normally distributed data [24,25].

### 2.2. Reported Incidents of Toxicity and Adverse Effects

Beta blocker toxicity and adverse effect incidents were examined separately for Belgium and Europe. Belgian data (2018–2023) were sourced from the Belgian Poison Centre, a national toxicovigilance service handling more than 60,000 calls annually [26]. Combination products were excluded to strengthen causal interpretation. The dataset contained exposure circumstances and patient demographics. European data were sourced from ADR reports provided by EudraVigilance, providing broader insight. EudraVigilance data from 2018 to 2023 were retrieved using standardized query parameters, specifying the substance name, the reaction group restricted to serious cases, and the sex and age group of the individuals. Serious cases are defined as: fatal, life-threatening, leading to hospitalization or prolonging an existing stay, causing permanent or significant disability/incapacity, resulting in congenital anomalies or birth defects, or other medically substantial conditions [27]. The analysis focused on the five most frequently reported beta blockers, including patient age and sex distribution, to compare patterns between regions.

### 2.3. Case Reports

Additionally, 27 propranolol-related fatality cases reported by the University of Antwerp Toxicological Centre between 2014 and 2023 were analysed, focusing on the presence of co-occurring co-exposures.

## 3. Results

### 3.1. Usage of Beta Blockers

**Figure 1:**
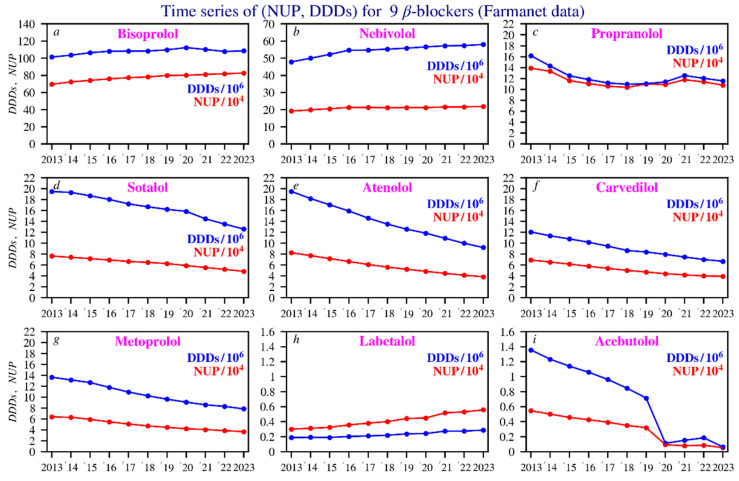
Time series of the NUP (in red) and DDDs values (in blue). The beta blockers are ranked by NUP according to their decreasing annual average. Scaling of the Y axis was performed for clarity. Subfigures: (**a**) bisoprolol; (**b**) nebivolol; (**c**) propranolol; (**d**) sotalol; (**e**) atenolol; (**f**) carvedilol; (**g**) metoprolol; (**h**) labetalol; (**i**) acebutolol.

Figure 1 presents the time series data for the DDDs and NUP spanning the 2013–2023 period of the nine most frequently prescribed beta blockers in Belgium. Detailed yearly values for NUP and DDDs per beta blocker can be found in Table S1 in the Supplementary Materials.

#### 3.1.1. NUP (Number of Unique Patients)

For most beta blockers, there is a slight decline in the number of unique patients over the 2013–2023 period, particularly for sotalol, atenolol, carvedilol, and metoprolol. For nebivolol and propranolol, the numbers remain relatively constant. In contrast, bisoprolol and labetalol show a slight upward trend over time. Table S2 in the Supplementary Materials presents the corresponding *p*-values for each beta blocker, as well as for total DDDs/NUP, DDDs, and NUP.

**Figure 2:**
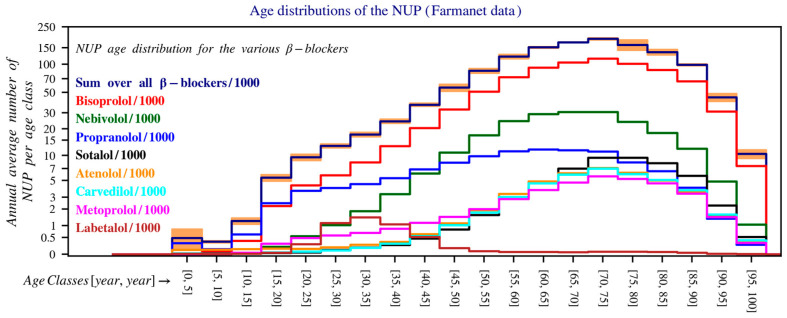
Age distribution of the number of unique patients in Belgium over the period 2020–2023. The *y*-axis is shown on a logarithmic scale and shows the average number of patients per year within each age group (0–5 years, 5–10 years, 10–15 years, etc.), calculated as the average of the total number of patients per age group over this period. For readability, the values have been divided by 1000. The bands on top of the histogram provide a measure for the uncertainty in the estimates for the bin values of the *y*-axis. The lower and upper bounds of these intervals are the mean minus and plus 1.96 times the standard deviation (Farmanet).

Between 2020 and 2023, propranolol (blue line) shows a different age profile, with a flattened peak in NUP, and a relatively even distribution across age groups (Figure 2).

Propranolol has a distinct age distribution pattern, with a pronounced peak in very young children (reflecting its use in haemangioma) and a much earlier decline, similar to the pattern seen in migraines. In contrast, the use of other beta blockers increases progressively across older age groups, consistent with indications such as atrial fibrillation and hypertension.

However, bisoprolol and other beta blockers show a clear peak in the 66–80 age group. The gender distribution is notable for propranolol and atenolol, where female users are significantly more prevalent than male users in nearly all age groups. Labetalol use is predominantly among females due to its role in pregnancy, with male users occurring mainly in older age groups (Figure S1, Supplementary Materials).

#### 3.1.2. DDD (Defined Daily Doses)

Decline for most beta blockers: This applies to sotalol, atenolol, carvedilol, metoprolol, and acebutolol, all of which show a clear downward trend. Acebutolol stands out due to its discontinuation since January 2023 [28]. Propranolol shows a relatively stable trend over time. To estimate average medication use per patient, the DDD is divided by the NUP. Propranolol, sotalol, carvedilol, and metoprolol show stable ratios, indicating consistent dosing. Bisoprolol, labetalol, and acebutolol show slight decreases, suggesting lower average doses. Nebivolol and atenolol show increases, pointing to higher dosing or longer treatment durations for remaining patients (Figure S2, Supplementary Materials). Trends over time in DDDs, NUP, and DDDs/NUP for each beta blocker were assessed using Kendall’s tau, a nonparametric test for rank correlation, implemented through Python-based statistical analysis. The corresponding *p*-values are provided in Table S1 in Supplementary Materials.

### 3.2. Reported Incidents of Toxicity and Adverse Drug Reactions

Reported incidents of toxicity and adverse drug reactions associated with beta blockers were analysed with a distinction made between data originating from Belgium and broader European sources. Belgian data were obtained from the Poison Centre and cover the period from 2018 to 2023. European data was derived from ADR (Adverse Drug Reaction) reports, providing a broader perspective on incidents across Europe. The analysis includes the five most frequently reported beta blockers, along with the gender and age distribution of affected patients, enabling a comparative assessment between Belgium and Europe.

#### 3.2.1. In Belgium According to the Belgian Poison Centre

Combination preparations were excluded to allow for more accurate attribution of toxicity. Among the reports, 307 involved combination products and 2827 involved single ingredient preparations. Reports of beta blocker poisonings fell slightly from 2018–2023. Bisoprolol caused most cases, followed by propranolol, nebivolol, sotalol, and atenolol. Women were affected nearly twice as often as men. Although women account for 56% of prescriptions, they represent 62% of poisoning cases.

A comparison between reports to the Poison Centre and the NUP shows that beta blocker users are mainly older adults (aged 60–80), while toxicological reports often involve children, usually due to accidents. Self-harm is more common among 10–20-year-olds, especially with propranolol, whereas overdose is more prevalent among older adults.

**Figure 3:**
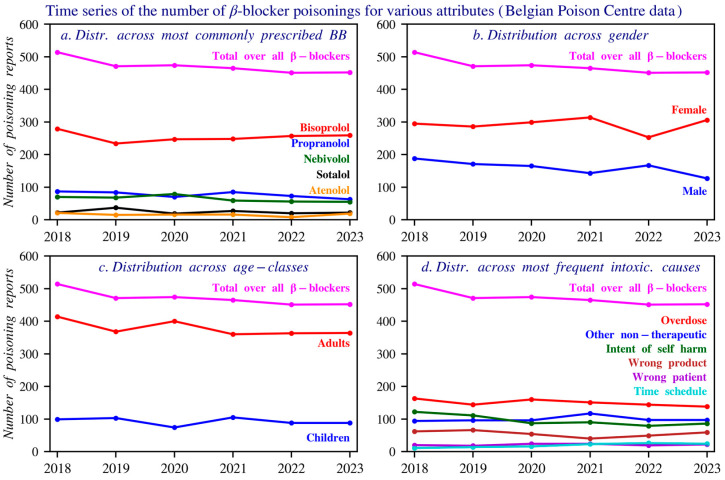
Time series of the number of reports, gender distribution, age distribution, and causes of beta blocker poisonings reported to the Poison Centre Belgium during 2018–2023. Individuals aged above 14 years are included. Panel (**a**) shows the distribution of the top five beta blockers involved in poisoning reports. Panel (**b**) presents the gender distribution across all reported poisonings. Panel (**c**) displays the age class distribution of individuals involved in these reports. Panel (**d**) illustrates the most frequently reported causes of poisoning, as documented by the Poison Centre.

**Figure 4:**
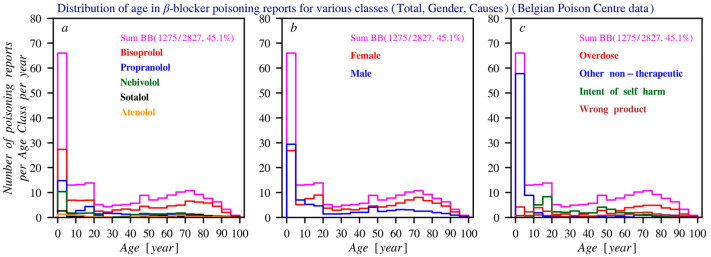
Histograms of the age distribution of the number of poisoning reports (average per year) according to the Poison Centre Belgium. In all three subfigures, the histogram drawn in purple represents the age distribution for the total number of reports per year (summed across all beta blockers). The following distributions are shown: (**a**) Distribution of the most frequently reported beta blockers. (**b**) Age distribution of the total number of reports. (**c**) Distribution of the most common causes of poisoning. For all histograms, the bin size is 5 years.

**Figure 5:**
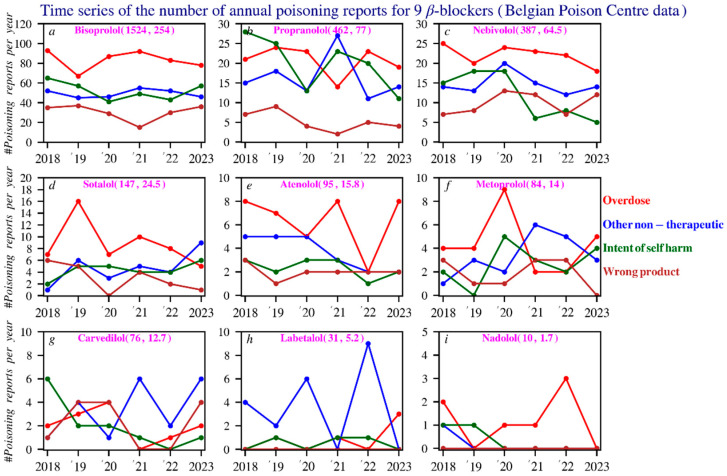
Poisonings in time series of the 9 beta blockers: An analysis of the primary causes. The numbers in purple show, respectively, the total number of reports and the average number of reports per year. Subfigures: (**a**) bisoprolol; (**b**) propranolol; (**c**) nebivolol; (**d**) sotalol; (**e**) atenolol; (**f**) metoprolol; (**g**) carvedilol; (**h**) labetalol; (**i**) nadolol.

The main non-therapeutic causes of poisonings are non-intentional overdose and intentional self-harm, with a higher frequency of self-harm involving propranolol compared to other beta blockers. The dataset used fixed category groups, which may allow some overlap, but each incident was recorded under a single primary cause. Figure 3 presents these data based on the total number of reports, gender, age distribution, and the most common circumstances over time. Figure 4 shows age histograms of beta blocker poisoning reports. Figure 5 gives an overview of the primary causes of poisonings for the 9 most commonly reported beta blockers.

To gain better insight into the relative poisoning frequency in relation to use, the number of beta blocker poisoning reports can be divided by the number of unique patients over a given period. This calculation assumes that the number of prescriptions reflects how widely each beta blocker is used in Belgium, and that poisoning reports are submitted in a similar way for all beta blockers. We also calculated the number of poisoning reports per total DDDs, using this as a second measure of exposure based on the idea that higher cumulative dosing provides more opportunities for misuse or accidental overdose. For this analysis, the overlapping years from the Belgian Poison Centre and Farmanet datasets (2018–2023) were used. Propranolol, acebutolol, labetalol, and nebivolol show comparatively higher poisoning ratios per user than the others beta blockers. When medication use (amount of DDDs) is taken into account, bisoprolol, propranolol, and nebivolol have the highest relative ratios. Propranolol stands out in both analyses: it causes relatively frequent poisonings per user as well as per amount of medication prescribed. Bisoprolol, in contrast, appears particularly risky when corrected for DDDs, meaning it causes more poisonings per unit of medication than other beta blockers (Figure S3, Supplementary Materials).

#### 3.2.2. In Europe According to ADR Reports

European ADR data (2018–2023), obtained via www.adrreports.eu, highlights serious adverse reactions reported for multiple beta blockers [27]. Only serious cases, as defined in the Methods section, were included in this analysis. Belgian reports are included in the European dataset, but their relative weight cannot be reliably determined because the system does not allow year-specific filtering, and many reports lack country identification.

Metoprolol accounts for the highest number of reports (7157; ~1193 per year), followed closely by bisoprolol (6642; ~1107 per year) and propranolol (2905; ~484 per year). Carvedilol and atenolol also show substantial numbers, with 2232 and 1892 reports, respectively. While several beta blockers appear to show temporal trends, only esmolol demonstrates a statistically significant increase.

Gender distribution indicates that most reports originate from women, except for bisoprolol, which is predominantly reported in men, while carvedilol shows an even split. Adults represent about 80% of cases, children about 4%, with propranolol standing out for its relatively high proportion of pediatric reports—responsible for roughly half of all serious adverse reactions in children.

**Figure 6:**
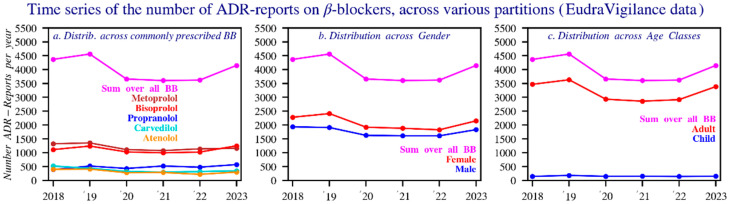
Time series of the number of reports of suspected adverse reactions for all beta blockers and the five main beta blockers by: (**a**) sex, (**b**) age, (**c**) during the period 2018–2023 (EudraVigilance). The dotted lines represent the average. In the dataset, an adult is considered 17 years or above.

Reaction categories classified by MedDRA show “cardiac disorders” as the leading group for most beta blockers. Propranolol is the exception, where the category “injury, poisoning, and procedural complications” is most frequently reported [29]. Figure 6 provides an overview of the results.

#### 3.2.3. Comparison Between Belgian and European Data

**Figure 7:**
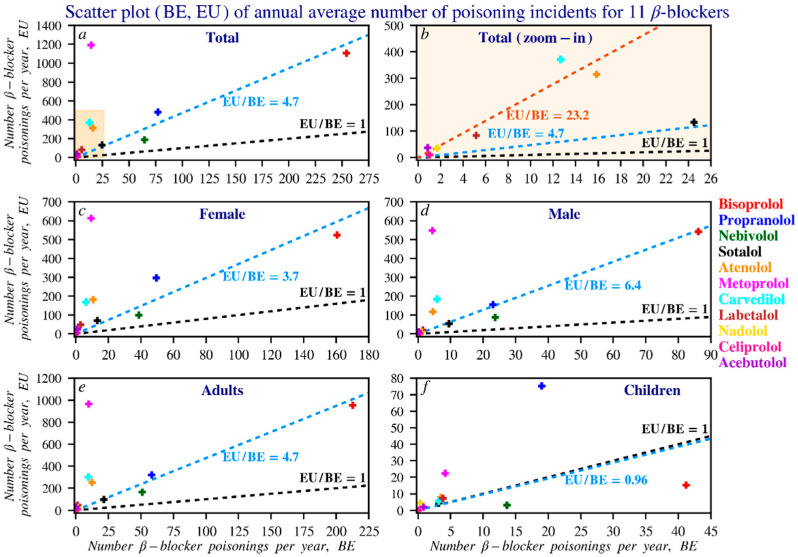
Scatterplot of beta blocker reports in Europe compared to those in Belgium. To improve readability, the counts were divided by 6 to better reflect the average number of reports per beta blocker per year. The black line, following the equation y = x, indicates where the number of reports in Belgium equals those in Europe. The blue line, y = ax, represents the ratio between EU and Belgian reports, where a = EU/BE. The larger the value of a, the steeper the slope of the line. Panel (**b**) is a zoomed-in view of the boxed area in panel (**a**), analysing beta blockers that are relatively infrequently reported in both Belgium and Europe. The red line in this panel is the regression line for the corresponding data points. Subfigures: (**a**) overall reports; (**b**) zoomed-in view of low-frequency reports; (**c**) female reports; (**d**) male reports; (**e**) adult reports; (**f**) children’s reports. In this dataset, an adult is defined as a person older than 17 years.

Comparing beta blocker reports between Belgium and Europe is challenging due to differences in reporting methods. In Belgium, causes are often known, and outcomes remain unclear. In Europe, both causes and outcomes are often missing. The lack of EU-wide sales data also makes it difficult to link usage trends to reported cases. Figure 7 provides an overview of the available data. Gender distribution is more balanced at the EU level than in Belgium, where propranolol ADRs are more often reported in women. Bisoprolol is frequently reported in both regions, while propranolol appears more often in child cases.

### 3.3. Case Studies

This section analyzes 27 fatal cases in which propranolol was detected, based on toxicological records from the University of Antwerp (2014–2023). Concentrations were measured via blood, serum, and (in one case) liver tissue, reported in ng/mL or ng/g.

**Figure 8:**
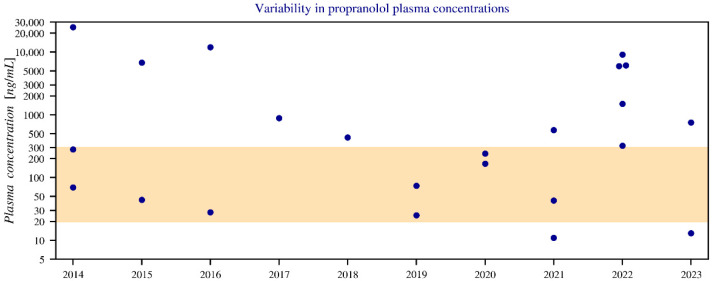
Annual variation in blood plasma concentrations of propranolol between 2014 and 2023, shown with a logarithmic *y*-axis. The graph displays only blood and serum data, while three values below the limit of quantification (LOQ) have been omitted. The coloured band represents the therapeutic boundaries of 20–300 ng/mL. Notably, outliers above the therapeutic range (such as in 2014, 2016, and 2022) show relatively few combinations with other medications, meaning the contribution of propranolol is greater. In contrast, cases below the therapeutic range (for example in 2021 and 2023) more often involve drug combinations, reducing the relative contribution of propranolol.

Concentrations ranged from below therapeutic levels to extremely high values (up to 25,000 ng/mL in plasma and 93,000 ng/g in liver), far exceeding the therapeutic range of 20–300 ng/mL. Propranolol was never the sole agent in intoxication cases. Toxic concentrations (>1000 ng/mL) and fatal ranges (4000–10,000 ng/mL), due to doses far beyond the standard doses (40–320 mg), emphasize the severity of overdose [30] (Figure 8).

**Figure 9:**
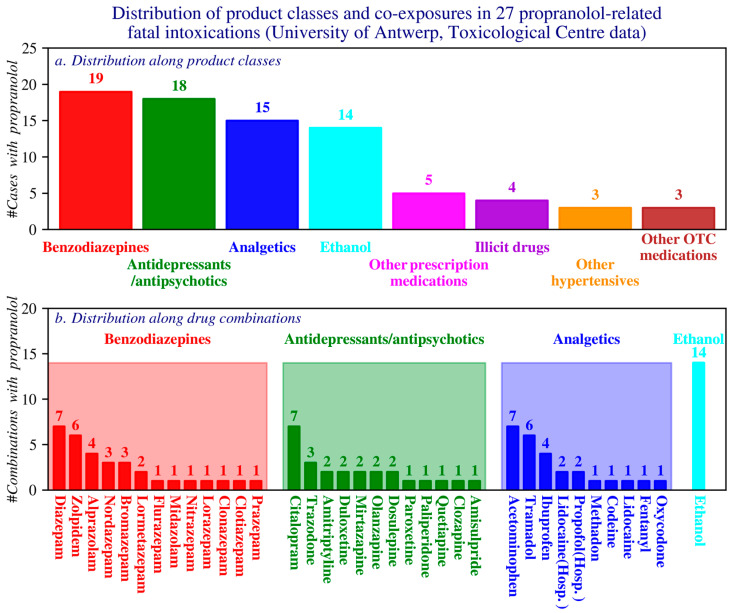
Distribution of case counts by product class (**a**) and by drug combinations within the four most frequently reported product classes (**b**). OTC: Over the counter products; Hosp: hospital products. (Toxicological Centre, University of Antwerp).

Common comedications comprised benzodiazepines, antidepressants/antipsychotics, analgesics, and ethanol; the most frequently used agents within these classes were diazepam, citalopram, and paracetamol, respectively (Figure 9).

## 4. Discussion

This study summarizes beta blocker use and toxicity patterns in Belgium and Europe. Belgian utilization is shaped by reimbursement rules, product availability, and newer agents, while Farmanet captures only reimbursed outpatient use. A stable DDDs/NUP ratio with declining absolute values likely reflects fewer users rather than dosing changes. Propranolol shows a stable DDDs/NUP ratio, indicating that the average dose per patient remains unchanged. Sotalol, carvedilol, and metoprolol display a similar pattern, with stable ratios despite declining user numbers. Bisoprolol, labetalol, and acebutolol show a slight decrease, suggesting lower average doses. In contrast, nebivolol and atenolol exhibit increasing ratios, pointing to higher average doses for nebivolol users and, for atenolol, higher doses or longer treatment durations among the remaining patients.

Bisoprolol, nebivolol, propranolol, sotalol, and atenolol are the most used beta blockers in Belgium. Sotalol and atenolol decline, bisoprolol and labetalol rise, and propranolol and nebivolol remain stable. Women more often receive propranolol and labetalol. Belgian poisoning data are harder to interpret due to limited demographic detail as only 45% of reports include age. Propranolol scores high on both poisoning ratios, indicating relatively frequent reporting both in absolute terms and when adjusted for prescribing volume. Despite the potential for disproportionate reporting, the propranolol data should be considered in clinical decision-making and monitoring.

At the European level, metoprolol generates the most reports, followed by bisoprolol and propranolol. Only esmolol shows a significant increase over time. EU data lack temporal and regional detail, hindering comparison with Belgium. Broader European datasets, including utilization figures, would allow for more reliable risk assessment. A key difference is that metoprolol is not among the top five beta blockers reports in Belgium, unlike in Europe. Data from DDDs and NUP show a decline in metoprolol use, possibly due to a shift toward other antihypertensives, like ACE inhibitors, calcium channel blockers, bisoprolol, and nebivolol. Nebivolol offers added benefits such as vasodilation and fewer sexual side effects [31].

Propranolol poisonings are relatively frequent compared to other beta blockers despite a lower prescription rate, suggesting a higher risk profile, especially among women and younger people at risk of self-harm. Based on international evidence, we expected to observe a similar pattern in Belgium, with propranolol appearing disproportionately often in poisoning reports despite its lower prescription volume. This expectation reflected both its pharmacological characteristics and its distinct prescribing profile compared with other beta blockers. As discussed in the introduction, propranolol’s disproportionate association with self-poisoning likely reflects its use in populations with elevated psychiatric vulnerability. Our findings are consistent with this broader pattern, showing an association between propranolol use and the presence of depressive symptoms and self-harm, but the results do not allow conclusions about causality. The association may partly reflect confounding by indication, as propranolol is more often prescribed for anxiety-related symptoms compared to other beta blockers, generating protopathic bias as early depressive symptoms present as anxiety. This challenge is well described in the broader literature on antihypertensives and mood disorders [32].

Interpretation is further complicated by other clinical contexts. Propranolol is also used chronically for migraine prophylaxis, and migraine is associated with approximately a twofold increase in suicidality [8]. Recent studies similarly caution against overinterpreting observational associations between beta blockers and psychiatric outcomes [33,34].

Despite these uncertainties, clinical relevance remains clear. The limited evidence for propranolol’s effectiveness in anxiety, combined with its reputation as a “safer” alternative to benzodiazepines, underscores the need for careful monitoring once treatment is initiated [10,18,33,35]. A brief note on the clinical positioning of propranolol is warranted, although our data offer only limited guidance for prescribing decisions. Comparable data from similar patients not receiving propranolol—particularly in psychiatric contexts—are lacking, and robust studies or randomized trials remain scarce.

Benzodiazepines, analgesics, antidepressants/antipsychotics, and ethanol dominate reported co-exposures, highlighting the need to manage polypharmacy and psychiatric risk. Health care workers play a key role in counselling, identifying drug interactions, limiting quantities dispensed, monitoring home supplies, ensuring safe disposal, and applying knowledge of intraclass toxicity differences to guide the choice of safer firstline agents [19,34]. In line with these responsibilities, several policy-oriented measures may further support safer use, including limiting package sizes and integrating smart pop-ups into prescribing software, as recommended in recent literature. Incorporation of such measures into future BCFI publications could strengthen national guidance and enhance their relevance for Belgian clinical practice [36]. Targeted prevention efforts for youth, older adults, and polypharmacy patients may help reduce harm. Although centred on Belgium, these findings are relevant across Europe.

### Study Limitations

A limitation is that Farmanet data include only reimbursed medications dispensed in public pharmacies and exclude non-reimbursed use. Age information was missing in more than half of Belgian poisoning reports from the Poison Centre, limiting demographic analyses. The categorization of intoxication causes in the dataset is based on fixed groups, and because some overlap between these groups is possible, assigning each incident to a single primary cause may not fully justified. Combination products were excluded, which reduced the dataset but allowed clearer attribution to one specific substance. At the European level, the inability to filter reports by year or region restricts temporal and geographic interpretation. Finally, utilization data were not available at the EU level, preventing correction of ADR frequencies for exposure. Strengthening reporting systems, improving cross-country comparability, and analysing vulnerable age groups and drug combinations—alongside reducing underreporting and clarifying ADR methods across Europe—remain essential to improving data quality and interpretability.

Despite these limitations, the study is strengthened by the breadth of data sources, combining national usage, poisoning, and ADR datasets from Belgium and Europe. This multisource, multi-country approach provides a uniquely comprehensive view of beta blocker utilization and toxicity patterns. Additionally, this study complements existing international literature and underscores how regional healthcare practices may influence propranolol intoxication patterns. The integrated nature of these data sources is unusual, as few countries offer similarly comprehensive linkage between utilization, poisoning, and toxicology records.

## 5. Conclusions

Taken together, these findings show that in Belgium, propranolol has a relatively higher association of poisoning compared with other beta blockers, particularly among younger patients and women. Co-use with psychoactive medications further amplifies this risk, underscoring the need for careful monitoring and targeted prevention. Strengthening the role of health care workers and patient education remains essential to reducing avoidable harm.

## Data Availability

The original contributions presented in this study are included in the article/Supplementary Materials. Further inquiries can be directed to the corresponding author.

## Supplementary Materials

The following supporting information can be downloaded at: https://www.mdpi.com/article/10.3390/pharmacy14020043/s1. Table S1: Beta blocker consumption and related data, 2013–2023 (Farmanet). Figure S1: Age and gender distribution of the number of unique patients in Belgium during the period 2020–2023, sorted by the highest total number of unique patients. The scale on the y-axis shows the average number of patients per year within each age group (0–5 years, 5–10 years, 10–15 years, etc.), calculated as the average number of patients per age group over this period. For readability, the values have been divided by 1,000. The total number of unique patients (NUP) is shown in black, while values for females are shown in red and those for males in blue. The dotted lines from left to right represent the 5%, 10%, 50%, 90%, and 95% quantiles, respectively. The solid line indicates the average total number of reports. The bands on top of the histogram provide a measure for the uncertainty in the estimates for the bin values of the y-axis. The lower and upper bounds of these intervals are the mean minus and plus 1.96 times the standard deviation. (Farmanet). Table S2: Trend analysis for DDDs/NUP, DDDs and NUP values in the period 2013–2023 (Kendall’s Tau rank correlation test implemented through Python-based statistical analysis). Figure S2: Defined Daily Dose (DDDs) per unique patient during the period 2013–2023 in Belgium. Figure S3: Intoxication ratios (number of reports per unique patient and number of reports per DDD per unique patient) for nine different beta blockers. To make a clearer distinction between beta blockers with striking ratios and those with comparable values, the figures have been split into separate sections. The ranges of the shaded areas in figures a and c correspond respectively to the ranges in figures b and d. The dotted lines represent the average intoxication ratios. Acebutolol stands out as an outlier (a), but due to the negligible number of reports, this gives a distorted impression in the graph.
