# Supplementary material for: Beta Blocker Intoxications in Belgium: A Data Analysis with Focus on Propranolol
Source: Pharmacy (Basel). 2026 Mar 4;14(2):43. doi: 10.3390/pharmacy14020043 (PMC13010693; doi:10.3390/pharmacy14020043)
Supplement: Supplementary file 1 [file pharmacy-14-00043-s001.zip › pharmacy-4101152-supplementary.pdf]

MDPI
pharmacy

Article

Beta blocker intoxications in Belgium: A data analysis with focus on propranolol

Brechje van den Boogaard [1], Maria van de Lavoir [1], Rani Robeyns [1], Celine Gys [1], Adrian Covaci [1], Hans De Loof [2*]

[1] Toxicological Centre, University of Antwerp, Universiteitsplein 1, 2610 Antwerp, Belgium
[2] Laboratory of Physiopharmacology, University of Antwerp, Universiteitsplein 1, 2610 Antwerp, Belgium

* Correspondence: hans.deloof@uantwerpen.be

Supplementary files

The raw data supporting the conclusions of this article will be made available by the authors on request.

Table S1. Beta blocker consumption and related data, 2013–2023 (Farmanet)

Year	Beta blocker	Number of unique patients (NUP)	Number of DDDs
2015	acebutolol	4,567	1,139,868
2016	acebutolol	4,268	1,059,730
2017	acebutolol	3,924	963,067
2018	acebutolol	3,511	846,682
2019	acebutolol	3,197	715,122
2020	acebutolol	943	112,427
2021	acebutolol	785	151,420
2022	acebutolol	864	185,430
2023	acebutolol	526	63,024
2013	atenolol	82,767	19,498,524
2014	atenolol	77,401	18,190,793
2015	atenolol	71,940	17,044,718
2016	atenolol	66,540	15,905,966
2017	atenolol	61,119	14,606,898
2018	atenolol	56,293	13,512,484
2019	atenolol	52,310	12,581,212
2020	atenolol	48,520	11,850,917
2021	atenolol	44,837	10,909,561
2022	atenolol	41,441	9,998,071
2023	atenolol	38,505	9,212,296
2013	bisoprolol	695,746	101,338,718
2014	bisoprolol	723,468	103,504,381
2015	bisoprolol	741,709	106,441,815
2016	bisoprolol	758,883	108,092,051
2017	bisoprolol	773,009	108,238,993
2018	bisoprolol	781,267	108,305,949
2019	bisoprolol	798,975	109,812,401
2020	bisoprolol	801,275	112,329,494

2021	bisoprolol	810,323	110,232,728
2022	bisoprolol	818,568	107,838,323
2023	bisoprolol	826,790	108,537,950
2013	carvedilol	69,265	12,067,461
2014	carvedilol	65,562	11,365,084
2015	carvedilol	61,774	10,798,640
2016	carvedilol	57,845	10,191,789
2017	carvedilol	53,938	9,468,764
2018	carvedilol	50,315	8,682,314
2019	carvedilol	47,247	8,392,958
2020	carvedilol	44,179	7,965,738
2021	carvedilol	41,990	7,471,191
2022	carvedilol	40,199	7,032,220
2023	carvedilol	39,318	6,681,881
2013	celiprolol	14,146	4,924,808
2014	celiprolol	12,994	4,469,780
2015	celiprolol	11,826	4,102,165
2016	celiprolol	10,704	3,736,642
2017	celiprolol	9,619	3,335,449
2018	celiprolol	8,539	3,030,604
2019	celiprolol	7,303	2,407,147
2020	celiprolol	5,974	2,188,522
2021	celiprolol	4,919	895,460
2013	labetalol	3,014	188,575
2014	labetalol	3,116	191,200
2015	labetalol	3,250	190,460
2016	labetalol	3,569	202,377
2017	labetalol	3,803	210,176
2018	labetalol	4,006	219,119
2019	labetalol	4,414	237,785
2020	labetalol	4,499	242,387
2021	labetalol	5,174	273,784
2022	labetalol	5,310	274,797
2023	labetalol	5,579	286,648
2013	metoprolol	63,818	13,653,428
2014	metoprolol	63,103	13,152,824
2015	metoprolol	59,255	12,671,045
2016	metoprolol	54,631	11,787,892
2017	metoprolol	50,810	10,928,571
2018	metoprolol	47,189	10,251,069
2019	metoprolol	44,728	9,625,314
2020	metoprolol	42,037	9,080,779
2021	metoprolol	40,518	8,593,094
2022	metoprolol	38,570	8,300,287
2023	metoprolol	36,651	7,846,012
2013	nebivolol	191,464	47,844,678
2014	nebivolol	198,868	49,985,478
2015	nebivolol	205,172	52,224,474
2016	nebivolol	212,685	54,665,996

2017	nebivolol	212,427	54,733,413
2018	nebivolol	211,349	55,303,486
2019	nebivolol	212,047	55,846,555
2020	nebivolol	211,847	56,594,697
2021	nebivolol	215,825	57,209,241
2022	nebivolol	216,029	57,374,584
2023	nebivolol	218,352	57,995,761
2013	pindolol	2,271	379,498
2014	pindolol	2113	346,865
2015	pindolol	1,994	326,697
2016	pindolol	643	80,176
2017	pindolol	8	488
2013	propranolol	138,767	16,162,183
2014	propranolol	133,162	14,283,413
2015	propranolol	116,136	12,494,142
2016	propranolol	110,454	11,794,140
2017	propranolol	105,927	11,176,282
2018	propranolol	103,617	10,938,482
2019	propranolol	109,939	11,031,566
2020	propranolol	108,889	11,370,744
2021	propranolol	117,689	12,542,801
2022	propranolol	113,610	12,021,114
2023	propranolol	107,770	11,553,326
2013	sotalol	76,582	19,487,182
2014	sotalol	74,264	19,322,154
2015	sotalol	71,823	18,702,573
2016	sotalol	69,302	18,026,860
2017	sotalol	66,700	17,209,976
2018	sotalol	64,847	16,678,011
2019	sotalol	62,818	16,215,596
2020	sotalol	59,048	15,850,069
2021	sotalol	55,516	14,483,471
2022	sotalol	52,111	13,519,151
2023	sotalol	48,517	12,595,104
**Total**		**14,719,553**	**2,509,207,674**

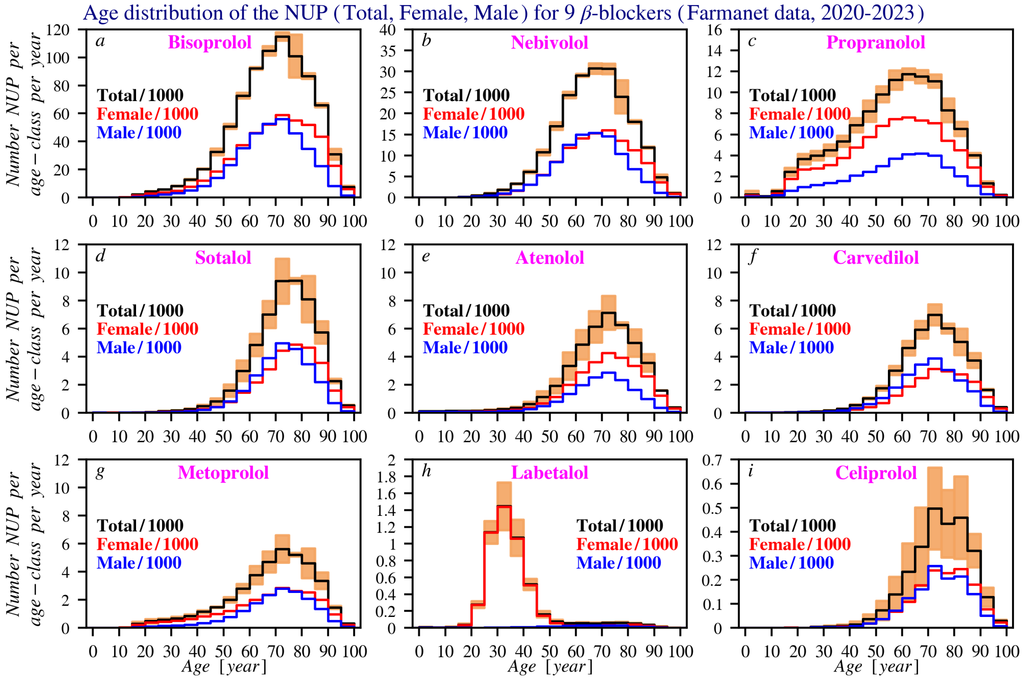

Figure S1. Age and gender distribution of the number of unique patients in Belgium during the period 2020–2023, sorted by the highest total number of unique patients. The scale on the y-axis shows the average number of patients per year within each age group (0–5 years, 5–10 years, 10–15 years, etc.), calculated as the average number of patients per age group over this period. For readability, the values have been divided by 1,000. The total number of unique patients (NUP) is shown in black, while values for females are shown in red and those for males in blue. The dotted lines from left to right represent the 5%, 10%, 50%, 90%, and 95% quantiles, respectively. The solid line indicates the average total number of reports. The bands on top of the histogram provide a measure for the uncertainty in the estimates for the bin values of the y-axis. The lower and upper bounds of these intervals are the mean minus and plus 1.96 times the standard deviation. (Farmanet).

Table S2. Trend analysis for DDDs/NUP, DDDs and NUP values in the period 2013–2023 (Kendall's Tau rank correlation test implemented through Python based statistical analysis).

**Beta blocker**	**DDDs/NUP ratio**	**P-value DDDs/NUP ratio**	**DDDs**	**P-value DDDs**	**NUP**	**P-value NUP**
bisoprolol	Significant decrease	0.00005	Significant increase	0.00571	Significant increase	$<10^{-6}$
nebivolol	Significant increase	0.00001	Significant increase	$<10^{-6}$	Significant increase	0.00159
propranolol	Non-significant decrease	0.16457	Non-significant decrease	0.21835	Non-significant decrease	0.12097
sotalol	Non-significant increase	0.54226	Significant decrease	$<10^{-6}$	Significant decrease	$<10^{-6}$
atenolol	Significant increase	0.00571	Significant decrease	$<10^{-6}$	Significant decrease	$<10^{-6}$

carvedilol	Non-significant increase	0.5426	Significant decrease	$<10^{-6}$	Significant decrease	$<10^{-6}$
metoprolol	Non-significant increase	0.44538	Significant decrease	$<10^{-6}$	Significant decrease	$<10^{-6}$
labetalol	Significant decrease	$<10^{-6}$	Significant increase	$<10^{-6}$	Significant increase	$<10^{-6}$
acebutolol	Significant decrease	0.00159	Significant decrease	0.00001	Significant decrease	$<10^{-6}$
Total	Significant decrease	0.00311	Significant decrease	0.00076	Non-significant decrease	0.08656

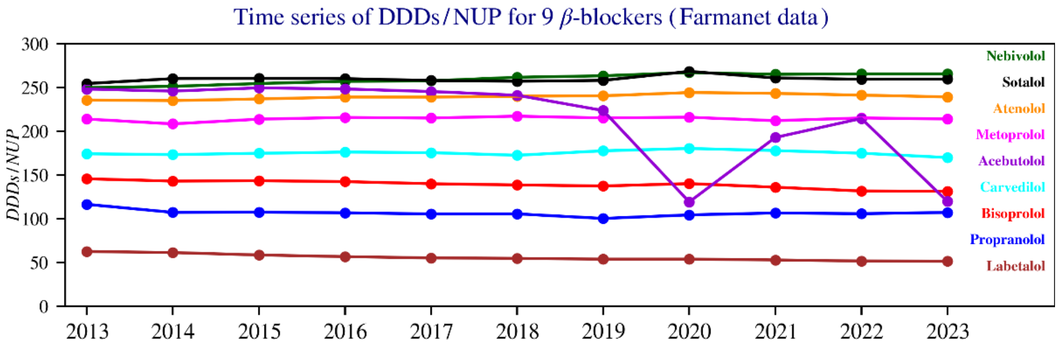

Figure S2. Defined Daily Dose (DDDs) per unique patient during the period 2013–2023 in Belgium

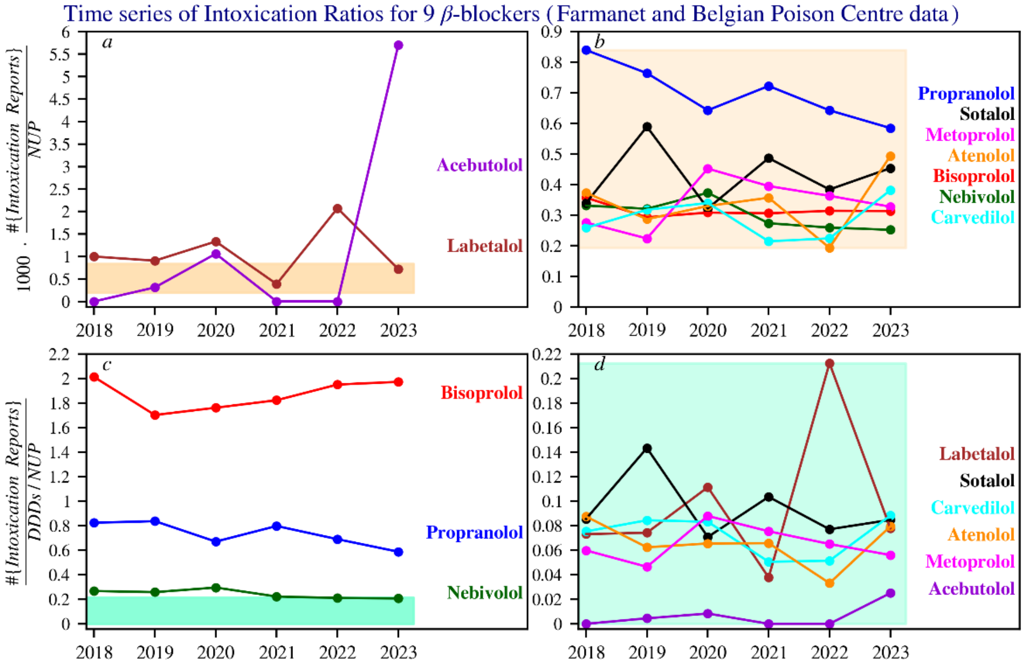

Figure S3. Intoxication ratios (number of reports per unique patient and number of reports per DDD per unique patient) for nine different beta blockers. To make a clearer distinction between beta blockers with striking ratios and those with comparable values, the figures have been split into separate sections. The ranges of the shaded areas in figures a and c correspond respectively to the ranges in figures b and d. The dotted lines represent the average intoxication ratios. Acebutolol stands out as an outlier (a), but due to the negligible number of reports, this gives a distorted impression in the graph.
